# Optical Polymer Waveguides Fabricated by Roll-to-Plate Nanoimprinting Technique

**DOI:** 10.3390/nano11030724

**Published:** 2021-03-13

**Authors:** Vaclav Prajzler, Vaclav Chlupaty, Pavel Kulha, Milos Neruda, Sonja Kopp, Michael Mühlberger

**Affiliations:** 1 Department of Microelectronics, Faculty of Electrical Engineering, Czech Technical University in Prague, Technicka 2, 168 27 Prague, Czech Republic; chlupvac@fel.cvut.cz (V.C.); nerudmil@fel.cvut.cz (M.N.); 2 PROFACTOR GmbH, Im Stadtgut D1, A-4407 Steyr-Gleink, Austria; Sonja.Kopp@profactor.at (S.K.); michael.muehlberger@profactor.at (M.M.)

**Keywords:** optical planar waveguides, roll-to-plate R2P nanoimprinting, UV-curable polymers, inorganic-organic hybrid polymer, optical losses

## Abstract

The paper reports on the properties of UV-curable inorganic-organic hybrid polymer multimode optical channel waveguides fabricated by roll-to-plate (R2P) nanoimprinting. We measured transmission spectra, refractive indices of the applied polymer materials, and optimized the R2P fabrication process. Optical losses of the waveguides were measured by the cut-back method at wavelengths of 532, 650, 850, 1310, and 1550 nm. The lowest optical losses were measured at 850 nm and the lowest average value was 0.19 dB/cm, and optical losses at 1310 nm were 0.42 dB/cm and 0.25 dB/cm at 650 nm respectively. The study has demonstrated that nanoimprinting has great potential for the implementation of optical polymer waveguides not only for optical interconnection applications.

## 1. Introduction

Integrated optical and photonic devices are playing an increasingly important role in optical communication networks, optical interconnections, optical data centers and the application of optical sensors [1]. The importance of these integrated optical devices grows even more due to the rapid widespread communication devices for smart cities data communications using internet applications in the Fiber-to-the-Home (FTTH) and Internet of Things (IoT) systems. Optical planar waveguides are the basic building blocks for the implementation of these optics and photonic structures and devices.

Up to now, various materials, including semiconductors such as silicon, silicon nitride, indium phosphide, III–V compound, silica, or optical crystals, e.g., lithium niobate, lithium tantalate, and rubidium titanyl phosphate, have been used for the fabrication of the optical waveguides [2]. Highly integrated optics and photonic devices fabricated from polymers have been subject to intense research in recent years [3]. The advantages of polymer materials compared above mention materials are easier fabrication processes and that lead to developed optics devices with significantly lower material and production costs.

Therefore, in the last decades, new types of polymers for optics and photonics applications were developed in many laboratories worldwide and some of them are commercially available [4,5]. These polymers include siloxane LIGHTLINK™ XP-6701A core, LIGHTLINK™ XH-100145 clad [6], UV-curable epoxy polymers Su-8, EpoCore/EpoClad [7,8,9], benzocyclobutene (Dow Chemical, Midland, MI, USA) [10], ZPU resin and polymers (ChemOptics Inc., Daejeon, South Korea) [11], inorganic–organic hybrid polymers OrmoClear^®^FX (micro resist technology GmbH), SUNCONNECT (Nissan Chemical Ltd., Tokyo, Japan) [12,13], UV exposure optical elastomer OE-4140 core, OE-4141 cladding (Dow Corning, Midland, MI, USA) [14], Truemode Backplane Polymer (Exxelis, Ltd., Washington, DC, USA) [15], polydimethylsiloxane Sylgard 184, LS-6943 [16,17] and etc. [4,5].

These new polymers have unique and excellent optical properties such as low optical losses at operating wavelengths (including infra-red spectrum), well-controlled and tuneable refractive indices, thermal and chemical resistance, mechanical, environmental stability and environmental-friendly fabrication processes etc. Optical planar waveguides are basic building blocks for the realization of optics and photonic devices and several different techniques for the fabrication of polymer optics waveguides devices have been reported. These fabrication methods include mask photolithographic technology and following wet etching process [9], photo-resist patterning combined with reactive ion etching [18], two-photon-polymerization [19], laser direct writing [8], electron beam writing [20], flexographic and inkjet printing [21], hot embossing process [22], photo-bleaching [23], etc. These methods involve many processing steps and can lead to long fabrication times and low yield. Therefore, technologies such as the stamping methods [24] are studied for mass production. These methods include roll-to-roll (R2R) nanoimprint lithography processes [25,26] and also roll-to-plate nanoimprinting. Theses roller-based technologies provides an opportunity to use polymers, which are leading material candidates for applications requiring inexpensive and mass productions [26]. These technologies are used for the fabrication of flexible electronics [27]. Further, possibilities for use in optics and photonics applications are now being studied.

In this work, we demonstrate the fabrication of the optical waveguides using a Roll-to-Plate (R2P) imprinting process. R2P imprint lithography is an imprinting process that employs a roller-mounded stamp (imprinting plate) and a rigid surface plate, where the substrate is mounted. The core of our R2P fabrication unit is a transparent cylinder that houses a UV-source in its center [28]. A distinct advantage of roller-based imprinting is the peeling-like separation process of imprinting plate and substrate. This facilitates the replication of complex structures as well as the imprinting on large areas. Roller-based imprinting has been proposed soon after the invention of imprinting and has been developed further in various variants like roll-to-roll or roll-to-plate [25,26]. In this paper, we focused on the R2P imprint replication of microscale structures. We optimized the fabrication procedure to the dimensions of the core waveguides 50 × 50 μm (width × height). These dimensions were used due to the standard dimensions of multi-mode optical waveguides used for optics communications. In many cases, it is more challenging to nanoimprint micro-sized features as compared to nano-sized features since the material, that has to be displaced during the imprinting process, has to be moved over larger distances (several tens of µm) as compared to nano-sized features, where the displacement takes place only over several 100 nm typically. Nevertheless, imprinting has distinct advantages also for those types of applications and feature sizes, like the direct patterning of functional materials and large area patterning as can be seen below.

## 2. Materials and Methods

Optical waveguides were fabricated using UV photopolymer Lumogen OVD Varnish 311 (BASF) for the cladding layer and UV-curable inorganic-organic hybrid polymer OrmoClear^®^FX (micro resist technology GmbH, Berlin, Germany) were used for the core layer. Properties of the UV photopolymer Lumogen OVD Varnish 311 (BASF) provided by the supplier are as follows: density 1.1 g/mL (22 °C), viscosity Brookfield (12 rpm) 75–120 mPa·s, the refractive index measured by ellipsometry 623.8 nm (He-Ne) *n* = 1.507. Properties of the OrmoClear^®^FX polymer are as follows: viscosity 1.5 ±0.3 Pa·s, the refractive index 1.555 (589 nm, exposure).

A nickel master mold was used for the fabrication of the polydimethylsiloxane (PDMS) master stamp. The PDMS master was made from Sylgard 184 (The Dow Chemical Company, purchased at ELCHEMCo Ltd., Zruč nad Sázavou, Czechia). A HoloPrint^®^ uniA6 DT nano imprinter (Stensborg A/S) was used for roll-to-plate (R2P) nanoimprinting (NIL) process.

The transmission spectra were measured with a UV-VIS-NIR spectrometer (UV-3600 Shimadzu, Shimadzu Deutschland GmbH, Duisburg, Germany) in the spectral range of 250–1750 nm. The refractive indices of the samples were measured by dark mode spectroscopy using the Metricon 2010/M prism-coupler system and the measurement was done at six wavelengths 532.0, 654.2, 846.4, 1308.2, 1549.1 and 1652.1 nm and set for transverse-electric (TE) polarisation. We used prism #200-P-4a with refractive index *n* = 2.1558 (λ = 632.8 nm) and the applied prism enabled the measurement of the range of effective refractive indices from 1.2 to 2.02 at wavelength λ = 632.8 nm.

The optical/geometrical inspections of the fabricated optical channel waveguides were carried out by an optical digital camera ARTCAMI equipped with optical head ZOOM Optics (Olympus Czech Group Ltd., Prague, Czech Republic) and the software QUICKFOTO (the Version 3.0, PROMICRA, Prague, Czech Republic) for the control of waveguides dimensions. The dimension of the fabricated stamps and optical waveguide channels were also inspected by the KEYENCE VHX-5000 microscope (KEYENCE INTERNATIONAL, Mechelen, Belgium).

The optical properties of the optical channel waveguides were determined using the cut-back method at wavelengths: 532 nm (laser Nd:YVO_4_), 650 nm (laser Safibra OFLS-5-FP-650), 850 nm (laser Safibra OFLS-6-LD-850), 1310 nm (laser Safibra OFLS-6CH, SLED-1310) and 1550 nm (laser Safibra OFLS-5-DFB-1550). The method is described in more detail below. The input light was coupled into the channel waveguide using a 50 μm core multi-mode fiber; a larger-core (62.5 μm) fibre was utilized as the output. The input/output fibres were precisely aligned to the channel waveguides by using high-precision 3-axis stages on the optical bench; the output light power intensity was detected by a Thorlabs PM200 optical power meter. A silicon detector (Thorlabs S151C) was used for the measurement at wavelengths of 532, 650, and 850 nm and an Indium gallium arsenide detector (Thorlabs S155C) was utilized for the measurement at 1310 and 1550 nm. Optical losses *α* were calculated using the following equation:

(1) $$\alpha =\frac{10\cdot \log\frac{P_{1}\left(W\right)}{P_{2}\left(W\right)}}{l_{1}-l_{2}\left(cm\right)},$$

where *l*_1_, *l*_2_ are the lengths of the channel waveguides and *P*_1_, *P*_2_ are output optical powers before and after cutting the waveguide, respectively. The accuracy of the optical measurement set-up is estimated to ±5%.

**Figure 1:**
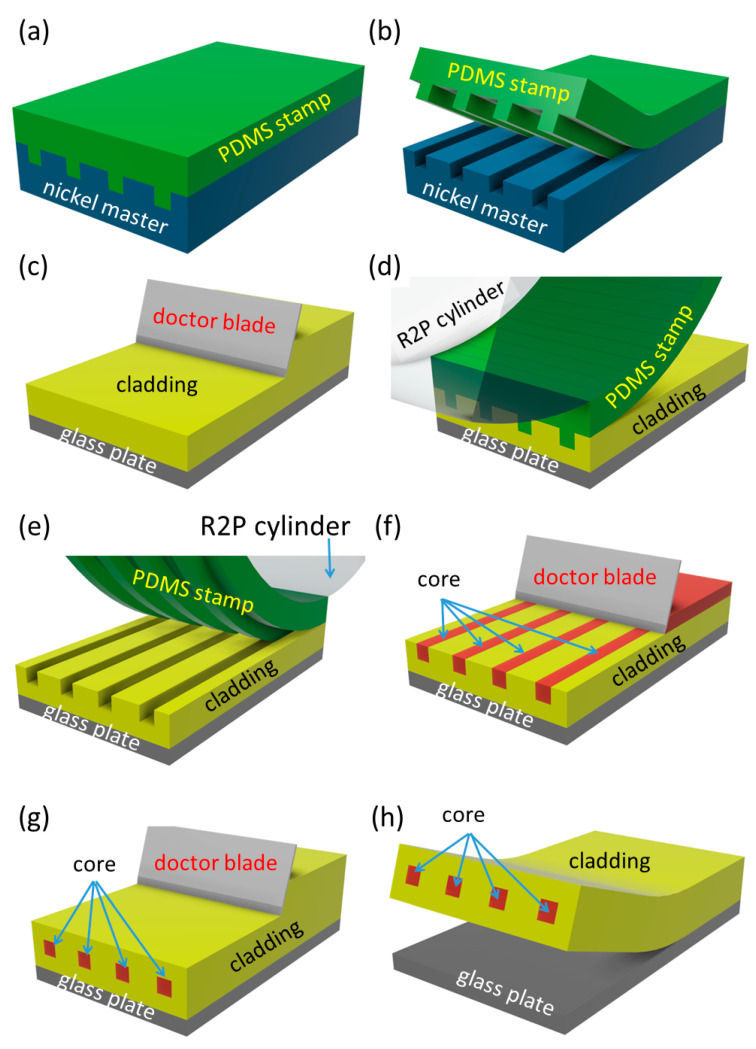
Fabrication process of the multimode optical waveguides using Roll-to-Plate imprinting, (**a**) fabrication PDMS (Sylgard 184) stamp layer, (**b**) separating stamp and Ni-mold, (**c**) fabrication of Varnish 311 UV layer, (**d**,**e**) fabrication of the U-groove into Varnish 311 UV substrate layer by R2P process, (**f**) fabrication of the OrmoClear^®^FX core layer into the U-groove Varnish 311 substrate, (**g**) fabrication of Varnish 311 UV cover cladding layer, (**h**) separating of waveguide structure from the glass substrate.

The fabrication process was as follows: The nickel master mold was used for the fabrication of the PDMS elastomer stamp. This nickel negative mold was made by the galvanoplastic process of a photoresist master produced by the photolithographic method. The mold was 8 cm long and had 12 channels with dimensions of 50 × 50 µm and 250 µm pitch between channels. PDMS stamps were fabricated from Sylgard 184 elastomer and elastomer was prepared by mixing the A and B agents in the ratio 10:1 and the mixture was stirred and then evacuated in a desiccator for 60 min. Then, the elastomer was poured onto the nickel mold and then the hardening process was done in the oven at 125 °C for 20 min (Figure 1a).

After cooling, the PDMS stamp was carefully torn off from the nickel mold (Figure 1b) and it was treated by separator SP-3 (ELCHEMCo Ltd., Zruč nad Sázavou, Czechia). Then the PDMS stamp was fixed on the cylinder of the R2P machine. After that, the polymer Lumogen OVD Varnish 311 cladding layer with a thickness of 500 μm was deposited onto the glass substrate by using the doctor blade technique (Figure 1c). The R2P machine was set properly before the start of the imprinting process. The most important parameters are the UV light intensity and the position of the cylinder height, and the imprinting speed. Because the cylinder height settings depend on the thickness of stamp and substrate, they have to be determined individually for each stamp/substrate thickness combination. The UV-light source in the R2P NIL tool uses 395 nm LEDs. After setting all parameters of the R2P machine, the PDMS stamp was imprinted into Varnish 311 UV photopolymer (Figure 1d,e). After that UV-curable inorganic-organic hybrid polymer OrmoClear^®^FX was deposited by doctor blading into the U-grooves from the Varnish substrate (Figure 1f). The OrmoClear^®^FX core layer was hardened by UV light @ 365 nm for 60 s (dose 100 mW/cm^2^). Next, a Varnish 311 UV photopolymer cover cladding layer was also fabricated by using the doctor blading technique (Figure 1g) and finally, the waveguide structure was torn off from the glass substrate (Figure 1h). Before separating the waveguides structure from the glass substrate, the Varnish 311 cover layer was hardened by a Mercury lamp (dose 1500 mJ/cm^2^). This process of the post UV curing finished hardening all other polymer layers (Lumogen OVD Varnish 311 substrate and an upper cladding layer, OrmoClear^®^FX core) which did not cure completely by the short bandwidth UV-light. This allowed the layers to bond together to create one integrated functional structure before the final UV curing step.

Finally, the optical waveguide end-facets for input/output optical fiber coupling were formed with a scalpel and polished. We used a three-step polishing process using polished (lapping) sheets with grit sizes of 3, 1, and 0.2 µm. This procedure reduced the length of the waveguides from 8 cm to approximately 5 ÷ 7 cm.

### 2.1. R2P Technology—Optimization Process

**Figure 2:**
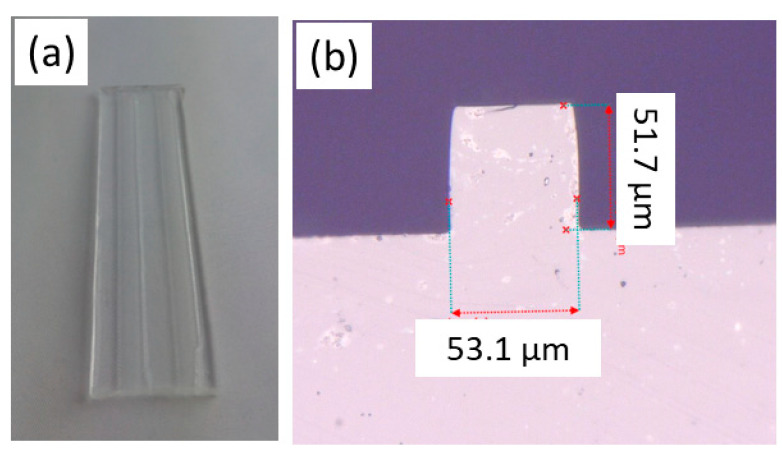
Picture of the Sylgard 184 stamp (**a**) camera picture whole stamp sample (total length of the PDMS stamp 8 cm), (**b**) microscope detail picture of the 50 × 50 μm channel.

One part of our research was also to optimize the fabrication procedure to achieve optical waveguides with dimensions 50 × 50 μm with pitch 250 μm and its homogeneity in the whole length of the waveguides. For the realization of optical channel waveguides with precision dimensions, it is important to fabricate a high-quality PDMS stamp, which is then copied into the UV polymer substrate layer using R2P imprinting. We fabricated this stamp by using a nickel master mold. An example of the fabricated stamp is shown in Figure 2. Figure 2a shows a picture whole PDMS stamp (length 8 cm) and Figure 2b shows a detailed KEYENCE VHX-5000 microscope picture of the one ridge channel. We expected to create channels with dimensions 50 × 50 μm with accuracy ± 5 μm. This picture proved the good quality of the stamp with sharp edges with required dimensions.

**Figure 3:**
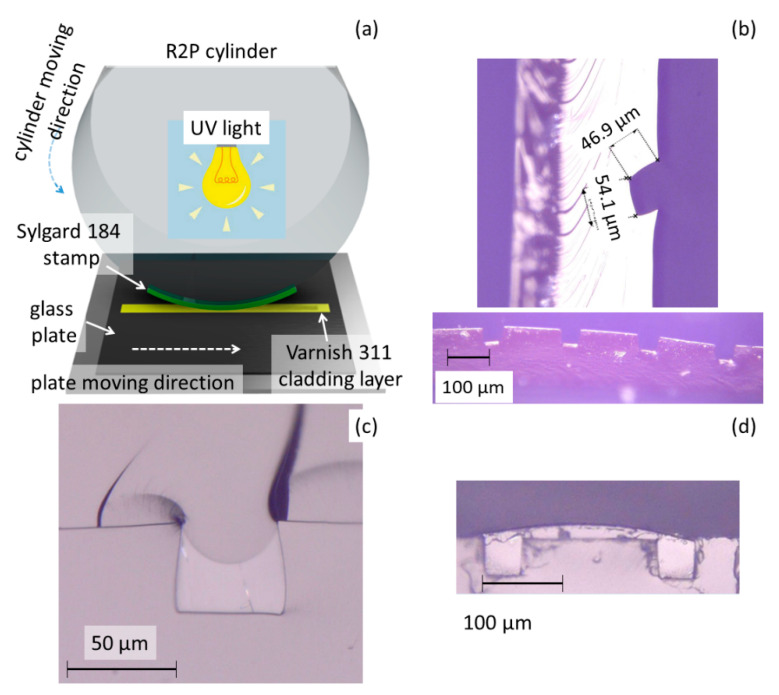
(**a**) Principle of the Roll-to-plate technology process. Results incorrectly set R2P process, KEYENCE microscope pictures (**b**) example of the fabricated U-groove channel in Lumogen OVD Varnish 311 by PDMS stamp, (**c**) example of the Lumogen OVD Varnish 311 U-groove fill in OrmoClear^®^FX core—channel is not filled correctly with the polymer core, (**d**) example of the Lumogen OVD Varnish 311 U-groove fill in OrmoClear^®^FX core—two channels are over-covered with the polymer core layer.

The dimensional precision of the fabricated optical channels waveguides depends on the distance between and glass plate and the R2P cylinder (see Figure 3a), which translates to an imprinting pressure, the speed of the plate moving under the R2P cylinder, and the UV curing dose applied not only during the R2P process, but also as post-processing UV curing.

In the case of the distance between the R2P cylinder and glass plate with the Lumogen OVD Varnish 311 cladding polymer was too high, the PDMS stamp was not imprinted into Varnish 311 polymer correctly and also the air bubbles were observed in the polymer layer. On the other hand, if the distance between the R2P cylinder and glass plate was too low the PDMS stamp was deformed due to the high pressure and the shape and dimensions of the channels were also not right (see Figure 3b, picture above). The channels were also not in the same right level position (see Figure 3b, picture below). In the HoloPrint^®^ (Dubai, United Arab Emirates) uniA6 DT, setting the roller height is done mechanically and the distance between substrate and roller can be up to 8 mm.

The UV curing process is the next important parameter and it depends on the exposure power of the applied UV source and the moving speed of the sample table. If the UV dose is higher than the curing process requires, then the polymer layer is hardened before the PDMS stamp was imprinted in the semi-cured material, therefore the channels have not the desired shape and dimensions. The case of lower UV dose expose caused an insufficient hardening of the material and the channels did not have again the correct shape the polymer was not solidified and could have liquid form after the process. In general, the UV intensity can be varied and a maximum dose of 100 mJ/cm^2^ at 6 m/min can be set. The minimum setting corresponds to 10 mJ/cm^2^ at 6 m/min or 30 mJ/cm^2^ at 2 m/min. The optimization process showed that the lowest UV light dose was suitable for the Lumogen OVD Varnish 311 cladding polymer.

The next parameter which has to be optimized is the moving speed of the plate with the substrate and the polymer cladding layer. A higher moving speed results in a lower dose of UV light and thus the UV polymer is less exposed. The substrate holder moving speed also affects the quality of the original PDMS stamp shape. At high speeds, wave-like artefacts and defects in the form of bubbles can be observed. Too small UV expose doses caused an insufficient hardening of the polymer layer and the channels did not have the correct shape—the material flowed even after the curing process. The used R2P machine allowed to set the moving speed from 0–8 m/min and completed tests showed that for applied polymers a speed of 2 m/min proved to be the most suitable.

The next step was to optimize the filling process of the core polymer into the U-grooves fabricated by R2P NIL. The core layer is made of the UV-curable inorganic-organic hybrid polymer OrmoClear^®^FX and for filling the core polymer into the U-groove we used the doctor blade technique. For this technique is important that the stamped layer was straight flat for easy squeegeeing the core polymer layer into U-groove channels. The next important thing was to choose an appropriate blade. Therefore, various blades were tested including plastic, rubber squeegees, and iron razor blades. The blades made of plastics and rubbers were too thick and the polymer core material was partly removed from the U-groove (see Figure 3c). Harder squeegees, in turn, left excess material between the channels (see Figure 3d). The best result was obtained when wiping using a sharp razor blade, which was sufficiently flexible and adapted to the wiped surface but did not remove core polymer from the channels. Therefore, this type of blade allowed homogeneous filling-up of the U-channels.

### 2.2. Stamp Modification

**Figure 4:**
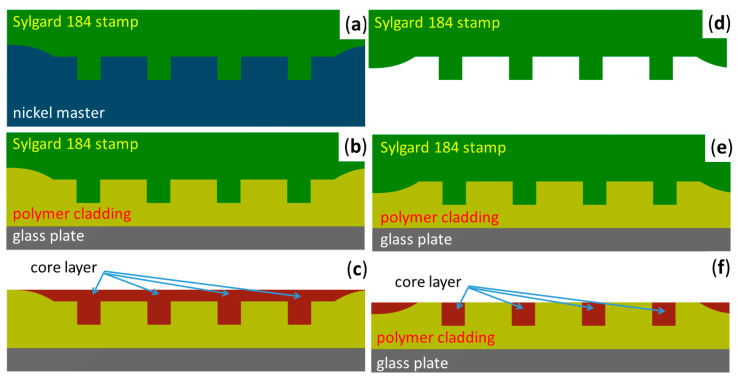
Illustrations of the Sylgard 184 stamp modification process. (**a**) Fabrication of the Sylgard 184 stamp using nickel master, (**b**) fabrication of the U-groove channels using Sylgard 184 stamp, (**c**) filing core polymer layer into U-groove channels, (**d**) modification of the Sylgard 184 stamp, (**e**) fabrication of the U-groove channels using modified Sylgard 184 stamp, (**f**) filing core polymer layer into U-groove channels.

The PDMS stamp in the function locations was made to the desired quality, but a spatial nonuniformity was formed on the left and right edges of the stamp (see Figure 4a) due to the shape of the Ni master. After the PDMS stamp was imprinted into the Lumogen OVD Varnish cladding layers, initially inequalities arose on the sides of the sample (see Figure 4b), which prevented the perfect wiping of the core layer when applying doctor blade technology and this layer remained on the cladding layer (see Figure 4c).

Due to this unevenness (see Figure 4b), we performed a PDMS stamp correction to refine the dimension and shape of the overall stamp. The correction was made after the separation of the PDMS stamp from the master nickel mold and Sylgard 184 polymer was added by a pipette to both edges of the stamp (see Figure 4d). Then the fabrication process of the U-groove into polymer substrate was done by R2P NIL (see Figure 4e). Figure 4f schematically shows filling U-groove channels after the doctor blading process using a modified PDMS stamp.

After cooling, the PDMS stamp was carefully torn off from the nickel mold (Figure 1b), and then it was treated by separator SP-3 to improve stamp life (ELCHEMCo Ltd., Zruč nad Sázavou, Czechia). If the separator SP-3 was not applied, we observed dimensional inhomogeneity and channel wall roughness in the U-groove Lumogen OVD Varnish 311 substrate after a few fabricated samples. In the case whereby the PDMS stamp was treated with the separator, we did not observe any defect until after more than ten produced samples.

## 3. Results

### 3.1. UV-VIS-NIR Transmission Spectroscopy and Dark Mode Spectroscopy

**Figure 5:**
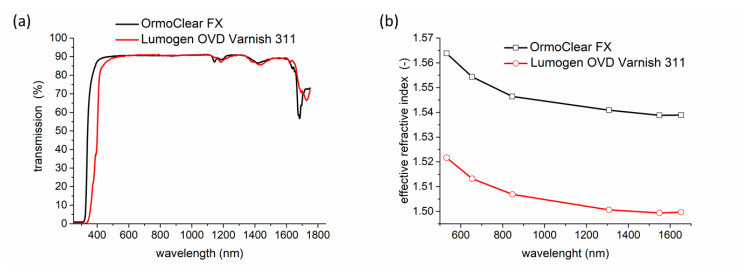
Properties of UV photopolymer Lumogen OVD Varnish 311 (BASF) and inorganic-organic hybrid polymer OrmoClear^®^FX (**a**) the transmission spectra, (**b**) the refractive indices.

The transmission spectra were measured with a UV-VIS-NIR spectrometer (UV-3600 Shimadzu, Shimadzu Deutschland GmbH, Duisburg, Germany) in the spectral range of 250–1750 nm. In Figure 5a, a comparison of the transmission spectra for Lumogen OVD Varnish 311 cladding layer and OrmoClear^®^FX core layer is given.

The refractive indices of the used polymers were measured by dark mode spectroscopy using six wavelengths: 532.0, 654.2, 846.4, 1308.2, 1549.1 and 1652.1 nm. The principle of the method has been already described in [12] and the measured results are shown in Figure 5b.

### 3.2. Dimensions of the Optical Channel Waveguides

**Figure 6:**
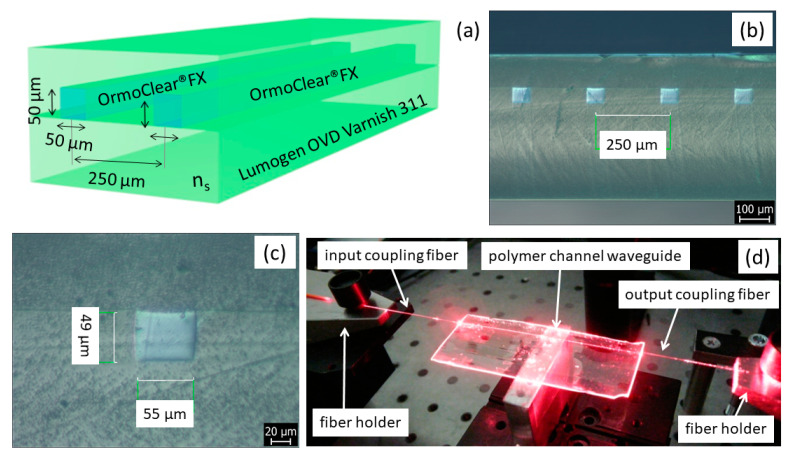
(**a**) Illustration of the optical channel waveguide, (**b**) the cross-sectional view of the four optical channel waveguides, (**c**) the detail view of the cross-section of a single optical channel waveguide, (**d**) photo of the measurement set-up with optical channel waveguide coupled with red light 650 nm.

The illustration structure of the optical channel waveguide with expected dimensions is shown in Figure 6a.

The final fabricated waveguide structures have twelve waveguides with 250 μm pitch with a length of around 8 cm. The optical ARTCAMI microscope picture of the four-channel waveguides is shown in Figure 6b,c, where Figure 6b shows the cross-sectional view of the four-channel waveguides and Figure 6c shows a detail of one channel waveguide. The figures show that channels have the required dimensions of 50 × 50 μm (height × width) with the channels pitch 250 μm with required accuracy dimensions ±5 μm. The figures also proved that channels had the required quality without any observable defects. Figure 6d shows a detailed picture of the optical losses measurement set-up, where the transmission of red light (650 nm) coupled via optical fiber into the channel waveguide is clearly visible.

### 3.3. Losses of the Optical Channel Waveguides

The optical losses were measured for eight samples where we measured eight optical channels in each sample (a total of 64 waveguides). The measurement started with determining the optical power *P*_1_ coming from a laser through the input coupling fiber and passing through the whole length of the measured channel waveguide to the output fiber connected with the optical power meter. Then the sample was cut and the output power *P*_2_ for the shortened sample was measured. The principle of the method has been previously reported in [17]. The results for the average measured values of the optical losses for eight waveguide channels are summarized in Table 1.

The results show that the highest values of the optical losses were at wavelength 1550 nm and the losses were higher than 1.10 dB/cm. Three samples (#1, #2 and #5) had higher optical losses than 1.30 dB/cm, therefore OrmoClear^®^FX polymer is not suitable material for realization optical waveguides operating at this wavelength. The lowest values of the optical losses were obtained at wavelengths 850 nm and the lowest average value was 0.19 dB/cm (sample #6). Very good results were also found for wavelength 1310 nm where average values of the optical losses were 0.38 dB/cm (sample #7). Optical losses measurement at visible spectrum for the red light at 650 nm showed that average values of the optical losses were also low and optical losses at a green light (532 nm) were a little bit higher than on red light (650 nm). All waveguides channels have lower values of the losses less than 1 dB/cm @ 532 nm except for the first two samples (#1, #2).

The lowest values of the optical losses were measured for sample #6 and the results measurement of the optical losses for eight channels are given in Table 2.

## 4. Discussion

### Optical Properties

Transmission spectra measurement shows that UV-curable inorganic-organic hybrid polymer OrmoClear^®^FX core layer is transparent in the whole measured spectral range from the visible to near-infrared spectrum (250–1750 nm). The measurement showed that this polymer has suitable properties as an optical waveguide core material and this result is also in good agreement with our previously published results in [12].

The measured values of the refractive indices *n* presented in Figure 5b proved that inorganic-organic hybrid polymer OrmoClear^®^FX has higher values of the refractive indices (1.5409@ 1308.2 nm) than UV photopolymer Lumogen OVD Varnish 311 layer (1.5006@ 1308.2 nm). Therefore, OrmoClear^®^FX hybrid polymer is a suitable candidate for the core layer with a combination Lumogen OVD Varnish 311 cladding layer. Our measured values of the refractive indices of OrmoClear^®^FX were also in good agreement with our previously published data and data provided by supplier micro resist technology GmbH [12]. OrmoClear^®^FX hybrid polymer used for the core layer has a higher value of the refractive index (*n* = 1.5409@ 1308.2 nm, 1.5388@ 1549.1 nm) compared to other optical polymers for example siloxane optical LIGHTLINK™ XP-6701A polymer (*n* = 1.505@ 1308.2 nm) or polydimethylsiloxane elastomers Sylgard 184 (*n* = 1.403@ 1308.2 nm) and NuSil Technology LS-6943 (*n* = 1.418@ 1308.2 nm). Therefore, and because of its excellent properties such as high transparency for visible and infrared light, high thermal, mechanical and chemical stability OrmoClear^®^FX hybrid polymer is a suitable candidate for advanced micro and nano-optical applications. In addition, OrmoClear^®^FX is also from chemical composition compatible with PDMS, making this material even more interesting.

The UV photopolymer Lumogen OVD Varnish 311 is a new material and therefore published data describing properties of this material are not available at this time. The refractive index value provided by the BASF supplier measured by ellipsometry is given only for red light *n* = 1.507 (He-Ne, 632.8 nm). Our measurement refractive index for a red light was *n* = 1.5132 (654.2 nm). This value is higher by 0.006 and the difference could be caused by using different fabrication procedures with comparison to the supplier fabrication process.

The average values of the optical losses for eight samples are depicted in Table 1 and results for the best sample (#6) presented for all measured channels are presented in Table 2. Optical communication systems, which use planar optical multimode waveguides with a geometric dimension of the waveguides channels of 50 × 50 μm, are optimized for operating wavelengths of 850 or 1300 nm. These types of waveguides are used for the distribution of the data in optical interconnection, short chip-to-chip, or board-to-board communications. In the last decade, the importance of these optical waveguides is growing due to the development of the next-generation optical interconnections communications systems in data-center, high-performance computers and etc. One of the most important properties of these waveguides is low optical losses, which have to be as low as possible and must not exceed the value of 1 dB/cm at operating wavelengths. Our measured data in Table 1 proved that presented waveguides have low optical losses and can be used in an optical communication system using wavelengths 850 and 1300 nm. For special short communications systems and optical sensors applications are also used optical wavelengths in the visible spectrum and our measurement proved that our waveguides with OrmoClear^®^FX hybrid polymer core layer and UV photopolymer Lumogen OVD Varnish 311 fabricated by R2P technology can be used also for operation on red light 650 nm. The average values of optical losses our waveguides were −0.43 dB/cm at 850 nm, −0.56 dB/cm at 1310 nm and −0.54 dB/cm at 650 nm respectively.

We compared our results with the waveguides presented with our previous works [9,17,29] and also with other authors’ [7,9,14,24,25]. We presented polymer multimode optical waveguides with the dimension of the channels 50 × 50 μm fabricated by the photolithography process. The waveguides were fabricated from epoxy polymer EpoCore/EpoClad and the average values of the optical losses for these waveguides were −1.85 dB/cm (650 nm), −0.60 dB/cm (850 nm) and −0.69 dB/cm (1310 nm), respectively [9].

Previously, we have already reported on flexible optical multimode elastomer polydimethyl-diphenylsiloxane channel waveguides (core layer LS-6943 NuSil, *n* = 1.4184 at 1311 nm, cladding Sylgard 184, *n* = 1.4030), where optical losses were lower than 0.45 dB/cm for four measured wavelengths 532, 650, 850 and 1310 nm) [17]. We have also presented elastomer waveguides with the same dimensions but here we used elastomer LS-6946 NuSil core layer and the average values of the optical losses were −0.76 dB/cm (650 nm), −0.59 dB/cm (850 nm), −0.56 dB/cm (1310 nm), and −1.93 dB/cm (1550 nm), respectively [29].

Bamiedakis et al. presented properties of multimode optical polymer waveguide for high-speed on-board optical interconnect fabricated from siloxane OE-4140 (core) and OE-4141 (cladding) material developed by Dow Corning and their waveguides exhibit low loss of approximately 0.04 dB/cm at 850 nm [14]. Choi et al. reported about properties of the multimode waveguide with the core size 50 × 50 μm and the core material of the waveguide was SU-8 and with Topas cladding. The measured propagation loss of the waveguide was 0.6 dB/cm at 850 nm [30]. Polymer-based thin-film foil waveguides fabricated by an industrial high-volume roll-to-roll embossing process were presented by Bruck et al. in [25] and they obtained propagation losses lower than 1 dB∕cm for a wavelength of 633 nm. The multimode waveguides fabricated by the stamping method by Kobayashi had the propagation loss of 0.06 dB/cm at a wavelength of 850 nm [24].

There are also papers that present single mode polymer optical waveguides for example Elmogi et al., 2016 [9]. They present the properties of two types of waveguides, which were fabricated by direct-write lithography and use epoxy EpoCore_5 and siloxane XP-6701A LightLink core layers. The dimensions of the core were about 5 × 5 μm and for the epoxy-based system, the average propagation losses are 0.49 dB/cm and 2.23 dB/cm at 1.31 μm and 1.55 μm, respectively. For the siloxane-based waveguides, the average propagation losses were 0.34 dB/cm and 1.36 dB/cm at 1.31 μm and 1.55 μm respectively [9]. Properties of the single-mode TE_00_-TM_00_ SU-8 rib waveguides with dimensions 5 μm wide and 0.7 μm high and with optical losses 1.36 dB/cm at 810 nm and 2.01 dB/cm at 980 nm, were presented in [7].

In this paper, we presented optical waveguides with optical losses lower or comparable with our previously presented results [9,17,29] and also comparable with results presented by Choi et al. [30]. Bamiedakis et al. presented an optical waveguide for high-speed applications with very low optical losses operating at 850 nm [14], but our applied R2P fabrication process allowed low cost and mass production. Our presented waveguides can also be used for the wider wavelength range from visible to infrared spectrum.

## 5. Conclusions

The paper has presented the properties of the planar optical waveguides fabricated by the roll-to-plate imprinting. PDMS stamps were fabricated from Sylgard 184 elastomer using a nickel master mold and the process was optimized for fabrication optical channels with dimension 50 × 50 μm and 250 μm pitch. These dimensions of the waveguides were chosen due to multimode fiber optics communications standards and increasing interest in new high-speed on-board optical interconnects used in data centers and in super high-speed computers.

The development of the fabrication process included optimizing the distance between the glass plate with the polymer layer and the R2P cylinder, the speed of the plate moving under the R2P cylinder and the UV curing dose applied during the R2P process and also post-processing UV curing. We also optimized the process of the deposition core layer by doctor blading technology and the sharp razor blade proved to be the most appropriate for our applied polymers.

The presented waveguides will allow the realization of a flexible optical waveguiding structure suitable for various applications. Due to their low optical losses, they would be useful for operations not only in the infrared spectrum at 850 and 1300 nm, which are the regions of major interest in optical interconnections but also in the visible spectral region which can be applied for optical visible communications or optical sensors. The paper has demonstrated that R2P NIL has great potential for the implementation of optical polymer waveguides not only for optical interconnection applications, and that the fabrication procedure allowed easy and mass production of the optical waveguide devices.

The next research will be focused to fabricate single-mode waveguides using R2P technology. We estimate dimensions for the single-mode waveguides from 0.5 µm up to 4 µm depends on the refractive index contrast for the applied materials and used transmitted wavelengths. The critical technology process for this purpose is to fabricate a high-quality PDMS stamp. Therefore, it is necessary to fabricate a high-quality master. We will use the laser beam writing process for the fabrication of the master stamp with the dimension of the single-mode waveguides and we also believe that it will be possible to then fabricated single-mode waveguides using R2P technology.

## Figures and Tables

**Table 1:** Average values of the optical losses measured by the cut-back method at wavelengths 532, 650, 850, 1310 and 1550 nm.

Sample	Length (cm)	Wavelengths (nm)				
		532	650	850	1310	1550
		Optical Losses (dB/cm)				
#1	5.71	−1.21	−0.96	−0.78	−0.79	−1.52
#2	5.27	−1.01	−0.82	−0.62	−0.70	−1.33
#3	6.76	−0.50	−0.51	−0.39	−0.53	−1.17
#4	6.76	−0.40	−0.47	−0.42	−0.54	−1.23
#5	7.06	−0.55	−0.41	−0.33	−0.52	−1.50
#6	7.06	−0.31	−0.25	−0.19	−0.44	−1.37
#7	5.80	−0.42	−0.43	−0.30	−0.38	−1.13
#8	5.80	−0.64	−0.48	−0.42	−0.54	−1.49
average value	6.28	−0.63	−0.54	−0.43	−0.56	−1.34

**Table 2:** Optical losses for sample #6 measured by the cut-back method at wavelengths 532, 650, 850, 1310 and 1550 nm.

Wavelength (nm)	Channel No.							
	1	2	3	4	5	6	7	8
	Optical Losses (dB/cm)							
532	−0.63	−0.49	−0.49	−0.56	−0.54	−0.52	−0.52	−0.66
650	−0.48	−0.38	−0.42	−0.38	−0.34	−0.36	−0.40	−0.54
850	−0.38	−0.31	−0.31	−0.30	−0.27	−0.32	−0.30	−0.40
1310	−0.58	−0.50	−0.50	−0.50	−0.47	−0.50	−0.51	−0.61
1550	−1.54	−1.50	−1.51	−1.44	−1.40	−1.54	−1.48	−1.59

## Data Availability

The data presented in this study are available on request from the corresponding author.
